# Functional Involvement of Signal Transducers and Activators of Transcription in the Pathogenesis of Influenza A Virus

**DOI:** 10.3390/ijms252413589

**Published:** 2024-12-19

**Authors:** Shasha Liu, Feng Qiu, Rongrong Gu, Erying Xu

**Affiliations:** 1 Key Laboratory of Fujian-Taiwan Animal Pathogen Biology, College of Animal Sciences, Fujian Agriculture and Forestry University, Fuzhou 350002, China; 2 Joint Laboratory of Animal Pathogen Prevention and Control of Fujian-Nepal, College of Animal Sciences, Fujian Agriculture and Forestry University, Fuzhou 350002, China

**Keywords:** influenza A virus, STATs, interferon, immune response

## Abstract

Signal transducers and activators of transcription (STATs) function both as signal transducers and transcription regulators. STAT proteins are involved in the signaling pathways of cytokines and growth factors; thus, they participate in various life activities and play especially critical roles in antiviral immunity. Convincing evidence suggests that STATs can establish innate immune status through multiple mechanisms, efficiently eliminating pathogens. STAT1 and STAT2 can activate the antiviral status by regulating the interferon (IFN) signal. In turn, suppressor of cytokine signaling-1 (SOCS1) and SOCS3 can modulate the activation of STATs and suppress the excessive antiviral immune response. STAT3 not only regulates the IFN signal, but also transduces Interleukin-6 (IL-6) to stimulate the host antiviral response. The function of STAT4 and STAT5 is related to CD4+ T helper (Th) cells, and the specific mechanism of STAT5 remains to be studied. STAT6 mainly exerts antiviral effects by mediating IL-4 and IL-13 signaling. Here, we reviewed the recent findings regarding the critical roles of STATs in the interactions between the host and viral infection, especially influenza A virus (IAV) infection. We also discuss the molecular mechanisms underlying their functions in antiviral responses.

## 1. Introduction

In 1994, Darnell found that signal transduction occurred when signal transducers and activators of transcription (STATs) proteins were phosphorylated; so, STAT proteins were named as signal transducers and activators of transcription (STATs). The molecular components of the STAT proteins have been identified for 40 years. STAT proteins are present in the cytoplasm prior to cytokine binding; they mediate cellular responses to cytokines and chemokines and play a key role in the antiviral immune response [1]. In the classical (Janus kinase) JAK/STAT signaling pathway, cytokines bind to the corresponding transmembrane receptors to activate the receptor-associated JAK kinase coupling, and subsequently, STATs are phosphorylated by JAK to form a dimer. Finally, STATs translocate through the nuclear membrane into the nucleus to specific sites to regulate the transcription and expression of relevant genes [2,3]. The JAK-STAT signaling pathway is able to transactivate and regulate a variety of cellular functions, including proliferation, migration, differentiation, and apoptosis, and it also has an important regulatory role in immune function [4].

The influenza virus is an enveloped, single-stranded, negative-stranded RNA virus belonging to the family *Orthomyxoviridae*. They are divided into four categories (types A, B, C, and D), of which influenza A viruses (IAV) can infect a wide variety of poultry and birds, animals, and human beings [5]. Its genome contains eight segments encoding at least 16 proteins [6,7]. Due to the genetic and antigenic variability of the two surface proteins, IAV is further classified into 18 hemagglutinin (HA) subtypes and 11 neuraminidase (NA) subtypes in animals and humans [8]. Following IAV infection of host cells, the cells induce the production of cytokines and chemokines through several different signaling pathways, thereby limiting the infection and enhancing the immune response to resist viral invasion [5]. IAV has also evolved the ability to antagonize the antiviral response in response to this. The non-structural protein-1 (NS1) is expressed at high levels in host cells and can induce the production of interferon (IFN) and modulate the host adaptation of IAV by inhibiting the expression of antiviral proteins and cytokines, such as STAT1 and interleukin 2 (IL-2) [9].

The STAT family is an important class of host proteins during IAV infection. In this paper, we investigated the roles and mechanisms of the STAT family in IAV infection and reviewed how STATs regulate the antiviral immune response and how IAV evades those immune responses.

## 2. The STAT Protein Family

### 2.1. Chromosomal Localization and Structure of STAT Proteins

**Figure 1:**
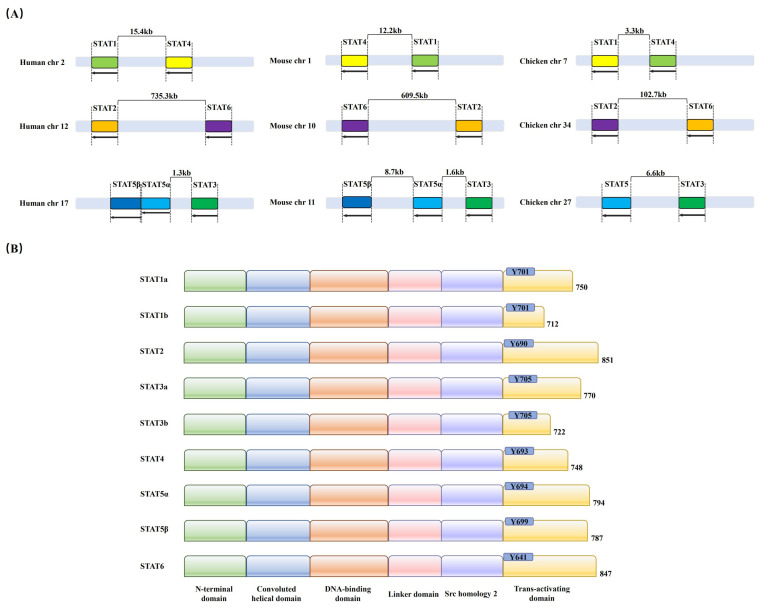
Arrangement of chromosomal localization and structure of STAT proteins. (**A**) The arrangement of IFITM gene clusters in humans, chickens, and mice. Arrows indicate the direction of transcription. Exons are represented as color, and introns are in gray. (**B**) The structure of STAT proteins. STAT proteins are composed of the following domains: *N*-terminal domain (ND), the convoluted helical domain (CCD), DNA-binding structural domain (DBD), linker domain (LD), Src homology 2 (SH2) domain, and carboxy-terminal transactivating domain (TAD). The STAT proteins consist of six members: STAT1, which possesses two splicing variants (STAT1a and STAT1b), STAT2 and STAT3, which also include two splicing variants (STAT3a and STAT3b), and STAT4, STAT5α, STAT5β, and STAT6.

In mammals, the STAT family contains seven members, STAT1, STAT2, STAT3, STAT4, STAT5α, STAT5β, and STAT6. The chromosomal localization of STATs is different. In human beings, STAT1 and STAT4 are both located on chromosome 2, while STAT2 and STAT6 are located on chromosome 12. STAT3, STAT5α, and STAT5β are all located on chromosome 17. In mice, STAT1 and STAT4 are located on chromosome 1, STAT2 and STAT6 on chromosome 10, and STAT3, STAT5α, and STAT5β on chromosome 11. Homologous STAT genes are also present in other species, including birds. However, the chicken STAT family has only six members, STAT1, STAT2, STAT3, STAT4, STAT5α, and STAT6, but no STAT5β. In chickens, STAT1 and STAT4 are located on chromosome 7, STAT2 and STAT6 on chromosome 34, and STAT3 and STAT5α on chromosome 27 (Figure 1A).

STAT proteins share a common structure that mediates intracellular signaling, and they are composed of the following domains: the *N*-terminal domain (ND), which mediates the oligomerization of STAT proteins; the convoluted helical domain (CCD); the DNA-binding domain (DBD); the linker domain (LD); the Src homology 2 (SH2) domain; and the carboxy-terminal transactivating domain (TAD) [10] (Figure 1B). The phosphorylation of tyrosine residues within the TAD domain plays a crucial role in STAT localization and activity. The tyrosine residues are STAT1-Y701, STAT2-Y690, STAT3-Y705, STAT4-Y693, STAT5-Y694, and STAT6-Y641 [11]. The phosphorylated STAT proteins translocate into the nucleus to initiate the transcription of target genes. The activated STATs are involved in different biological processes, such as immune adaptation, tissue repair, lipogenesis and apoptosis, and tumor formation [12].

### 2.2. STAT Proteins’ Induction in Different Bird Species

Avian influenza poses a significant threat to chickens in comparison to ducks, which exhibit greater resistance to most strains of the virus. To date, the underlying mechanisms remain incompletely elucidated. For instance, the highly pathogenic avian influenza virus (HPAIV) H5N1 causes severe infection in chickens at near complete mortality. The underlying molecular differences in host response may be that the expression of STAT3 was downregulated in chickens but was upregulated or unaffected in ducks during H5N1 infection [13]. Also, the proteomics analysis of the proteome and phosphoproteome datasets of domestic ducks (Anas platyrhynchos domesticus) infected with H5N1 revealed that the induction of STAT proteins, such as STAT1, STAT3, STAT5β, and STAT6, established a protective antiviral immune response [14]. It has also been reported that STAT1, STAT3, and STAT4 were upregulated in ducks, but showed no response in chickens during H5N1 infection. In addition, STAT TF binding sites were only enriched in the promoters of the duck DEGs and not in orthologous chicken promoters [15]. Together, those may be the reasons why ducks infected with most avian influenza viruses are asymptomatic or only cause mild clinical symptoms.

### 2.3. Biological Processes Involving STAT Proteins

**Figure 2:**
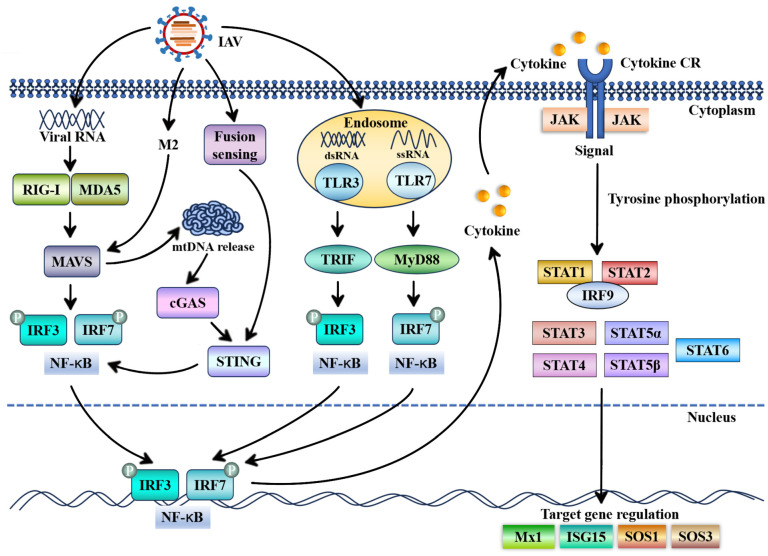
Schematic diagram for STATs against influenza A virus (IAV) infection. Host-specific pathogen recognition receptors (PRRs), such as RIG-I, MDA5, TLR3, and TLR7, recognize conserved components of IAV and then transmit signals to corresponding adaptor proteins, such as MAVS, TRIF, and MyD88. These adaptor proteins subsequently activate a series of transcription factors, such as IRF3, IRF7, and NF-κB, triggering the expression of cytokines, including IFNs. The influenza virus fusion peptide of hemagglutinin and M2 protein evokes STING pathways to induce IFNβ-expression. The interaction between cytokines and cytokine receptors (CRs) leads to JAK signal transduction, which activates transcription factor STATs. Activated STATs are transferred into the nucleus to regulate the expression of IFN-stimulated genes (ISGs). The dashed blue line represents the nuclear membrane of the cell.

STAT proteins are key transcription factors mediating cellular function, cell localization, nuclear transport, and other activities closely linked to transcription processes [16,17,18]. STAT proteins respond to external stimuli and rapidly stimulate gene expression, primarily regulating cytokines in immune cells. The primary pathway involved is the cytokine-activated JAK-STAT pathway. During IAV infection, the classical pathway is as follows: after IAV invades the host, the viral conserved components, known as pathogen-associated molecular patterns (PAMPs), are recognized by host pattern recognition receptors (PRRs), including retinoic acid-inducible gene I (RIG-I), melanoma differentiation-associated protein 5 (MDA5), toll-like receptor 3 (TLR3), and TLR7 [19]. This recognition initiates a cascade of signals transmitted to the downstream adaptor proteins, (mitochondrial antiviral signaling protein) MAVS, (myeloid differentiation primary response gene 88) MyD88, or TIR-domain-containing adapter-inducing interferon-β (TRIF), which subsequently recruits and activates transcription factors IRF3, IRF7, or NF-κB to enter the nucleus to enhance the expression of IFNs and pro-inflammatory cytokines. In addition, the STING pathway evokes the production of IFNs. The influenza virus M2 protein promotes the release of mitochondrial DNA (mtDNA) in a MAVS-dependent manner. Subsequently, the cytoplasmic mtDNA is recognized by the DNA sensor cGAS, which triggers STING-dependent IFN-β expression [20]. The IAV fusion peptide of hemagglutinin interacts with STING to induce IFN-β [21]. The cytokines bind to their respective receptors, activate the JAK-STAT pathway, and ultimately induce the expression of ISGs [22]. A schematic illustration of IFN-mediated JAK/STAT activation is shown in Figure 2.

Unphosphorylated STATs (U-STAT) also form dimers, enter the nucleus, and regulate transcription [23]. U-STAT3 was the first well-characterized U-STAT in mammalian cells. In the absence of IFN treatment, the ISGs, such as OAS1, OAS2, OAS3, and Mx1, were significantly upregulated in cells with stable STAT1 expression, indicating that U-STAT1 may enhance the expression of certain ISGs [24]. In the absence of IFN treatment, U-STAT2 constitutively binds to numerous IFN-activated promoters and facilitates their basal regulation [25].

The phosphorylated STAT1 is involved in the signal transduction of type I IFN (IFN-α and IFN-β), type II IFN (IFN-γ), type III IFN (IFN-λ), and IL-27, and it regulates cell proliferation, differentiation, and apoptosis [26,27]. The phosphorylated STAT2 effectively mediates transcriptional responses to type I IFN and type III IFN and inhibits or promotes tumor development by regulating type I IFN [28,29]. STAT3 is known as a multifunctional molecule in the STAT family. The phosphorylated STAT3 plays crucial roles in the regulation of various cellular biological processes by mediating the expression of downstream genes, such as cell proliferation, survival, differentiation, migration, angiogenesis, inflammation, and autophagy [30,31]. For instance, STAT3 and IL-6 constitute a key carcinogenic pathway and play an important role in the progression of many solid tumors, such as breast cancer [32,33]. In the STAT family, STAT4 is the only one with tissue-specific expression properties, being expressed constructively in lymphoid cells and induced in monocytes and macrophages [34]. STAT4 specifically mediates IL-12 signal transduction, induces the production of IFN-γ, and promotes the differentiation of T helper type 1 (Th1) [35]. STAT5 mediates the biological functions of the gamma cytokine family and the development of Tregs, playing an important role in the malignant progression of tumors and diseases [36,37]. STAT6 is a Th2 inducer stimulated and activated by IL-4 and IL-13; it is associated with the pathophysiology of various allergic diseases such as asthma, atopic dermatitis, and food allergies [38,39]. Meanwhile, STAT6 is involved in the development of various tumors, such as lymphoma and solitary fibroadenoma [40].

## 3. Antiviral Roles of STAT Proteins and Their Underlying Mechanisms in Influenza A Virus Infection

Recent studies have reported immune response interactions between STAT proteins and a variety of viruses, including the Zika virus (ZIKV), Sudan virus (SUDV), and African swine fever virus (ASFV) [41,42,43]. Some STAT proteins have also been studied under the influence of IAV, but the exact properties of these STAT proteins are unknown. During IAV infection, different viral proteins and thousands of host factors, including proteins and lncRNAs, were expressed to regulate the activation of STATs, some of which directly or indirectly affect the functional roles of STATs or alter cell metabolism by regulating the immune system. This study emphasizes the role of STAT proteins in the pathogenesis of IAV from the perspective of the mechanism.

### 3.1. Regulatory Roles of STAT1 and STAT2 During Influenza A Virus Infection

STAT1 and STAT2 play crucial roles in the immune response against IAV infection. Host cell PRRs sense the influenza virus, activate downstream signals, and then induce the expression of cytokines. The cytokines bind to their corresponding receptors. Type I IFN is recognized by IFN-α/β receptors (IFNAR), including IFNAR1 and IFNAR2; IFN-γ is recognized by the receptors IFNGR1 and IFNGR2 [44], while the recognition of type III IFN relies on the dimer IFNLR1 and IL-10 receptor 2 (IL-10R2) [45]. The binding of cytokines to specific receptors activates receptor-associated JAK1 or tyrosine kinase 2 (TYK2), which promotes the phosphorylation of STAT1 or STAT2 [46,47]. Phosphorylated STAT1 and STAT2 (P-STAT1, P-STAT2) assemble into STAT1-STAT2 heterodimers or STAT1-STAT1 homodimers, subsequently recruiting IFN regulatory factor 9 (IRF9) to form the IFN-stimulated gene factor 3 (ISGF3) complex. This complex is ultimately translocated to the nucleus. There, it interacts with IFN-stimulated response element (ISRE) to upregulate the expression of different proteins [17,46,48], such as the transcriptional expression of IFN-stimulated genes (ISGs), ultimately activating the cellular antiviral response and inhibiting viral replication [49,50].

After IAV infection, STAT1 can be regulated from various perspectives, thereby affecting the cellular antiviral response. For instance, the pandemic influenza A (H1N1) 2009 virus (pH1N1) infection exhibited dose- and time-dependent activated STAT1, and high-dose infection induced robust and earlier activation [51]. Additionally, it has been reported that the expression of Ten-Eleven Translocation 2 (TET2), a methylcytosine dioxygenase, was reduced in IAV-infected THP-1 cells and A549 cells. This led to the demethylation of the STAT1 gene, which ultimately inhibited the expression of the STAT1 protein [52]. Moreover, spleen tyrosine kinase (Syk), a non-receptor tyrosine kinase, triggers the activation of STAT1 in an IFN- independent manner via the RIG-I/mitochondrial antiviral signaling protein (MAVS) signaling pathway during the early stage of IAV infection [53]. But in the later stage, Syk inhibits the expression of type I IFN, thereby negatively regulating the activation of STAT1 [54]. In addition, P27Kip1, also known as Cdkn1B (Cdk inhibitor 1B), can significantly strengthen IAV-induced activation of STAT1 and promote the expression of key ISGs, such as IFITM3, MX1, and ISG15 [55].

Furthermore, some non-coding RNAs (ncRNAs) have been found to activate STAT1 during IAV infection. A long non-coding RNA (lncRNA) called RIG-I-dependent IAV-upregulated noncoding RNA (RDUR) was found to activate STAT1, which in turn positively regulated the IRF3/IFNs axis to trigger ISG expression [56,57]. Another type of ncRNA, called lncRNA-155, is encoded by the MicroRNA 155 host gene (MIR155HG). After lncRNA-155 is transcribed, it hydrolyzes to produce 22-nt RNA, known as miR-155. When induced by IAV infection, miRNA-155 can promote STAT1 phosphorylation by regulating downstream signaling molecules of IFN [57,58]. Together, ncRNAs can activate STAT1 through several mechanisms.

Activated-STAT1 is essential for the establishment of the antiviral response. In STAT1 knockdown A549 cells, the expression of ISGs, such as MX1, ISG15, and OAS2, decreased significantly after IAV infection. Similar results were obtained from the lungs of WSN-infected STAT1^−/−^ mice. Disruption of STAT1 Y701 phosphorylation suppresses antiviral response both in vitro and in vivo [53]. STAT1 is required to protect IAV-primed effector cells from NK cell-mediated deletion, as evidenced by the finding that STAT1^−/−^ CD4 T cells primed by IAV are susceptible to NK cell attack [59]. In addition to directly activating the antiviral effect, STAT1 can also inhibit IAV-induced inflammation. For instance, STAT1 plays a role in regulating the expression of neutrophil gelatinase-associated lipocalin (NGAL) or Lcn2 in influenza-induced myocarditis, leading to neutrophil infiltration caused by myocarditis [60]. Recent studies found that type I IFNs bind to the promoter of macrophage colony-stimulating factor receptor (M-CSFR) via STAT1. M-CSFR promoter DNA was found in STAT1 pull-down chromatin immunoprecipitation (ChIP) assays. Those indicated that STAT1 was critical for inducing the expression of M-CSFR. And the IAV-infected STAT1^−/−^ mice displayed an obstruction of emergency monocyte production and enhanced bacterial clearance [61] (Table 1).

STAT2 is essential for the establishment of the antiviral response to IAV infection. The STAT2 knockdown A549 cell showed enhanced virus replication and higher viral titer. Disruption of STAT2 phosphorylation at Tyr690 suppressed the expression of ISGs such as RSAD2, ISG15, and OASL [62] (Table 1). STAT2^−/−^ mice displayed excessive inflammation, viral burden, and increased morbidity after infection with influenza virus [63]. In a recent study, Bucciol et al. revealed that patients with complete STAT2 deficiency had significantly increased rates of infection with severe viruses, particularly severe influenza pneumonia, severe COVID-19 pneumonia, and herpes simplex virus type 1 (HSV-1) encephalitis [64]. In conclusion, STAT2 deficiency underlies severe viral diseases characterized by excessive inflammation due to impaired responses to type I IFN in the initial phase of infection.

IAV evolved multiple strategies to evade the IFN response by inhibiting the activity of STAT1 or STAT2 [65,66,67]. The IAV PB2 protein reduces cellular sensitivity to IFNs, weakening the activation of STAT1/STAT2 by targeting JAK1 at lysine 859 and 860 for ubiquitination and degradation [68]. IAV-induced host SOCS1 and SOCS3 block STAT1 from entering the receptor site [67,69,70]. In addition, Guanylate-binding proteins (GBPs) were significantly upregulated during IAV infection. GBP knockout enhanced IAV-induced IFN expression and phosphorylation of STAT1 and STAT2, indicating that the induced GBP7 enhanced IAV replication by suppressing innate immune responses [71]. The IAV NS1 protein serves as a critical determinant of virulence and exhibits multifaceted functions, such as inhibition of the processing of type I IFN precursor mRNA (pre-mRNA), disruption of the ubiquitin system, and regulation of the host fitness of influenza viruses [8]. In addition, the IAV NS1 protein directly upregulates the level of transcriptional readthrough (TRT) to decrease STAT1/STAT2 expression and cell viability [72]. To make matters worse, these strategies eventually lead to serious disease in the host. Life-threatening pulmonary influenza can be caused by inborn errors of type I and III IFN immunity. Further analysis indicated that STAT2/IRF9 potentially substituted for the role of ISGF3 and mediated antiviral immune responses in the existence of a STAT1-independent type I IFN signaling pathway [73]. A child with inherited IRF9 deficiency disrupted the activation of ISGF3 trimers (STAT1/STAT2/IRF9) and suffered from severe influenza pneumonitis, indicating that the IRF9- and ISGF3-dependent type I and III IFN pathways are essential for suppressing IAV replication [74]. Co-infection with influenza virus and Staphylococcus aureus repressed type I IFN-mediated STAT1 phosphorylation, ultimately providing benefits for influenza virus replication. Most fatal influenza cases are a result of secondary pneumonia caused by bacterial super-infections [75]. Together, the interaction between STAT1, STAT2, and viruses is complex, and the mechanism between STAT1, STAT2, and IAV mentioned in this paper is only a small part.

### 3.2. Functional Involvement of STAT3 in Regulation of Influenza A Virus Infection

STAT3 is required for antiviral activity against IAV. More than 40 different polypeptide ligands, including the interleukin-6 (IL-6) family, IL-10, Bv8 (also known as prokineticin 2 [Prok2]), and other cytokines, are known to activate STAT3. STAT3 was upregulated in various cell types from IAV patients, including naive T cells, cytotoxic CD8+ T cells, NKs, and DCs [76]. IAV-induced IFN activated STAT3 and provided negative feedback by promoting cell proliferation and survival, enabling the expression of genes with anti-inflammatory properties [77]. High-dose pH1N1 infection induced robust and earlier STAT3 activation [51]. Interestingly, the researchers found that STAT3 activity was negatively correlated with the pathogenicity of IAV strains. For instance, the HPAI H5N1 strain impaired the phosphorylation of STAT3 Y705; yet, the reduction was even more marked in the low-pathogenic seasonal H1N1 strain [73]. Consistently, overexpression of STAT3 significantly increased HPAI H5N1 virus replication in chicken cells [74]. The survival of mice with post-influenza pneumococcal pneumonia was improved by the STAT3/Bv8 axis [78]. IAV replicated to significantly higher titers in STAT3^−/−^ MEF cells and STAT3 knockdown A549 cells [79]. Moreover, the disruption of STAT3 Y705 phosphorylation in A549 cells enhanced IAV replication. Compared with WT mice, STAT3^Y705F/+^ mice were more susceptible to IAV infection. In the mechanism, disruption of STAT3 Y705 phosphorylation aggravates viral pathogenesis by excessive production of type I and III IFNs in vivo [80].

However, influenza viruses have evolved strategies to evade the STAT3 antiviral response. IAV led to impaired phosphorylation of STAT3 through IL-6-independent stimulation of SOCS3 overexpression [81]. Li et al. showed that IAV-upregulated host protein Angiopoietin-like 4 (ANGPTL4) enhanced pulmonary tissue leakiness and damage by a direct IL6-STAT3-dependent pathway [82]. In addition, there are a number of ncRNAs that can suppress STAT3 expression. For instance, STAT3 is regulated by miR-141 in vivo; the author showed that the expression of STAT3 was significantly decreased in cells transfected with pre-miR-141 during IAV infection [83]. MiR-4485 inhibited H1N1-induced severe pneumonia by suppressing STAT3 expression; the authors found that silencing STAT3 reversed the effects of miR-4485 downregulation on H1N1-induced cell injury [84]. Moreover, cells transfected with a putative miRNA put-miR-34 mimic showed a reduction in STAT3 expression and its phosphorylation [85]. In conclusion, STAT3 can effectively resist the invasion of IAV, and the virus has evolved strategies to escape the limitations of STAT3.

### 3.3. Important Roles of STAT4 During Influenza A Virus Infection

STAT4 is a member of the STAT family. After cytokines such as IL-12, IL-23, and type I IFN bind to specific receptors, STAT4 is phosphorylated, dimerized, and translocated to the nucleus to regulate gene expression [35]. STAT4 is also a prototypical Th1 transcription factor that regulates the expression of genes associated with pathogenicity in Th17 cells [86]. Typical Th1 polarization requires IFN-α-mediated synergistic activation of STAT1 and STAT4 to maximize the induction of the “master regulator” Th1 transcription factor (T-bet) [87,88].

IAV promotes Th1-like CD4 Th cell responses, and IAV virus-triggered STAT4 activation in CD4 Th cells maximizes the antiviral effects of Th1 effector-triggered T-bet and promotes a robust T-bet-dependent antiviral effector program. In addition, the amounts of NP-specific IFN-γ-producing Tc1 cells and HA-specific IFN-γ-producing Th1 cells were decreased, as shown in the spleens of STAT4^−/−^ mice during IAV infection. IFN-γ-independent STAT4 mediates the production of the T2 component during influenza virus infection [89]. It has also been shown that STAT4 was generally activated in a type I IFN-dependent manner, thereby regulating the IFN-γ response in IAV-infected NK cells [90]. Currently, research on the relationship between STAT4 and IAV is limited, and investigations into the underlying mechanisms are insufficient. Moving forward, there remains significant potential for exploration in this area.

### 3.4. Roles of STAT5 in Influenza A Virus Infection

STAT5 consists of two isoforms, STAT5α and STAT5β. When cytokines such as IL-2 and IL-3 bind to their respective receptors, STAT5 is phosphorylated and forms homo- or heterodimers between the two isoforms. The activated STAT5 dimer translocates into the nucleus to regulate gene transcription, such as cyclin D, serine/threonine kinase Pim-1, and Janus kinase-binding protein (JAB) [18,91]. In addition, STAT5 is a major regulator of energy and amino acid metabolism in CD4+ Th cells [92]. For instance, Ikaros zinc finger transcription factor (Eos) has emerged as a key regulator of CD4+ T cell differentiation. In Eos-deficient CD4+ Th2 cells, the IL-2/STAT5 axis and its downstream Th2 gene targets are one of the most significantly downregulated pathways [93].

Sustained high levels of phosphorylated STAT5 in plasmablasts was observed in a prior H1N1 virus challenge study in humans [94]. Via the mechanism, STAT5 can be activated via depletion of membrane-associated guanylate kinase with inverted domain structure-1 (MAGI1) to induce a strong anti-IAV response [95,96]. The role of plasmablasts expressing high levels of pSTAT5 warrants further investigation, but the abundance of plasmablast pSTAT5 following immunization and its correlation with the human adenovirus 5-based oral influenza vaccine tablet (VXA-A1.1) may protect humans against the H1N1 virus challenge [95]. In summary, the interplay between STAT5 and viruses remains inadequately understood, with numerous avenues for further investigation in the future.

### 3.5. Regulatory Roles of STAT6 Against Influenza A Virus Infection

STAT6, as a member of the STAT proteins, is principally activated by IL-4 and IL-13 [39]. Activated STAT6 exhibits diverse biological functions in the immune system and plays a crucial role in various biological processes of B cells, significantly influencing their development, activation, and tolerance [97]. For instance, activated STAT6 is able to modulate viral-induced type 2 inflammation to promote the expression of CCL17 and CCL22 [98]. The alteration of STAT6 activity can precipitate allergic reactions, tumorigenesis, autoimmune disorders, and other related conditions [99].

**Table 1:** The role of STATs against influenza viruses and their mechanisms.

STATs	Effect on Influenza Virus	Mechanism of Action	References
STAT1/STAT2	Type I IFN inhibited IAV replication	STAT1 or STAT2 is phosphorylated during IAV infection and STAT1-STAT2 heterodimers are formed by modulation of type I IFN	[39,41,42]
	ISGs inhibited IAV replication	STAT1 and STAT2 promote the expression of key ISGs	[43,44,49]
STAT3	Type I IFN inhibited IAV replication	STAT3 is upregulated in IAV-infected cells by modulation of type I IFN	[71,74]
	ISGs inhibited IAV replication	Phosphorylated STAT3 promoted the production of early ISGs, such as MX1, OASL1, and OAS3	[75]
STAT4	IFN-γ inhibited IAV replication	STAT4 triggers CD4Th cells to produce IFN-γ, leading to anti-influenza virus effects	[83,84]
STAT5	Immune response inhibited IAV replication	STAT5 modulates immune response to IAV infection by transduction of IL-2	[85,87]
STAT6	Inflammatory cytokines and chemokines inhibited IAV replication	STAT6 is upregulated and exerts antiviral effects on inflammatory cytokines and chemokines	[95,98,99]

Recent research found that STAT6 could be upregulated during IAV infection. Stimulation with IL-4 increased phosphorylation of STAT6 in PR8 (A/Puerto Rico/8/1934, H1N1)-infected bone marrow-derived macrophages (BMDM) [100]. STAT6 may play an important role in IAV-induced lung inflammation and can serve as a key therapeutic target. For example, Cyclosporin A (CsA) is able to modulate macrophage polarization through the IFN-γ/STAT1 and IL-4/STAT6 signaling pathways to modulate IAV-induced inflammatory responses [101]. However, STAT6 in fine bronchial exocrine cells can play a role in the inflammation caused by IAV infection [102]. STAT6 plays roles in the inflammatory cytokine and chemokine response to IAV infection. The mRNA levels of IL-13 were remarkably attenuated in STAT6^−/−^ mice compared to wild-type (WT) mice after IAV infection [103]. Compared with bone marrow-cultured mast cells (BMCMC) from WT mice after A/WSN/33 infection, BMCMC from WT or STAT6^−/−^ mice had a ~50% reduction in the production of IL-6 [104]. However, unlike the IAV-infected STAT1^−/−^, STAT2^−/−^, or STAT3^Y705F/+^ mice, the STAT6^−/−^ mice infected with IAV exhibited no significant changes in bodyweight loss and increased the lung viral load compared to IAV-infected WT mice. In addition, the increase in collagen staining also showed no significant difference between STAT6^−/−^ mice and WT mice at 21 and 49 d after IAV infection [103]. Together, the mechanism by which STAT6 responds to IAV infection remains to be explored.

In summary, although there have been new advances in the study of STATs in response to IAV infection, we need to conduct extensive and in-depth studies on STATs to pave a new path for the development of influenza vaccines and other medical research.

## 4. Conclusions

The STAT family, as transcription factors of various cytokines, play crucial roles in the host antiviral immune response. This review focuses on the role of STATs in IAV infection and their mechanisms. STATs collaborate with various cytokines and proteins to play important regulatory functions in immune responses. STATs are important targets for the research of vaccines and antiviral drugs and provide a rich experimental basis and research reference for the study of the anti-influenza virus immune response. However, the study of several STATs is still incomplete and needs to be explored more deeply. For example, the roles of STAT4, STAT5α, STAT5β, and STAT6 in IAV infection and their mechanisms should be further investigated. For instance, whether the various mechanisms of action that have been proven for STAT1, STAT2, or STAT3 can also be applied to STAT4, STAT5α, STAT5β, and STAT6 should be investigated. The mechanism underlying IAV-infected STAT6^−/−^ mice with no significant changes in bodyweight loss and increased lung viral load will be of great interest.

In turn, IAV has evolved various abilities to antagonize the antiviral response and is able to establish infection by negatively regulating the JAK/STAT signaling pathway to inhibit the expression of type I IFN. In addition to IAV, most pathogenic RNA viruses have also evolved strategies to evade the antiviral innate immune response [105]. Also, flaviviruses have developed several highly distinct mechanisms to block IFN signaling. NS5 of flaviviruses mediates proteasomal degradation of STAT2 to evade IFN signaling, whereas ZIKV in the genus Flavivirus degrades human STAT2, negatively affecting IFN signaling in a proteasome-dependent manner [106].

## Data Availability

No new data were created or analyzed in this study. Data sharing is not applicable to this article.
